# Model for predicting early and late-onset postoperative pulmonary complications in perioperative patients receiving neuromuscular blockade: a secondary analysis

**DOI:** 10.1038/s41598-023-32017-5

**Published:** 2023-03-31

**Authors:** Cristian Aragón-Benedí, Pablo Oliver-Forniés, Ana Pascual-Bellosta, Sonia Ortega-Lucea, José Manuel Ramírez-Rodriguez, Javier Martínez-Ubieto, Research Group in Anaesthesia, Resuscitation, and Perioperative Medicine of Institute for Health Research Aragón (ISS Aragón), Cristian Aragón-Benedí, Ana Pascual-Bellosta, Sonia Ortega-Lucea, Javier Martinez-Ubieto, Luis Alfonso Muñoz-Rodríguez, Guillermo Pérez-Navarro, Natividad Quesada-Gimeno, Mariana Hormigón-Ausejo, Raquel de Miguel-Garijo, Teresa Jiménez-Bernadó, Berta Pérez-Otal, Carmen Heredia-Coca

**Affiliations:** 1 Department of Anesthesia, Resuscitation and Pain Therapy, Miguel Servet University Hospital, 50009 Zaragoza, Spain; 2 Department of Anaesthesia, Resuscitation and Pain Therapy, Mostoles General University Hospital, Mostoles, Madrid, Spain; 3 Department of Surgery, Faculty of Medicine, University of Zaragoza, Zaragoza, Spain; 4 Institute for Health Research Aragón (IIS Aragón), Zaragoza, Spain

**Keywords:** Risk factors, Medical research

## Abstract

Pulmonary complications continue to be the most common adverse event after surgery. The main objective was to carry out two independent predictive models, both for early pulmonary complications in the Post-Anesthesia Care Unit and late-onset pulmonary complications after 30 postoperative days. The secondary objective was to determine whether presenting early complications subsequently causes patients to have other late-onset events. This is a secondary analysis of a cohort study. 714 patients were divided into four groups depending on the neuromuscular blocking agent, and spontaneous or pharmacological reversal. Incidence of late-onset complications if we have not previously had any early complications was 4.96%. If the patient has previously had early complications the incidence of late-onset complications was 22.02%. If airway obstruction occurs, the risk of atelectasis increased from 6.88 to 22.58% (p = 0.002). If hypoxemia occurs, the incidence increased from 5.82 to 21.79% (p < 0.001). Based on our predictive models, we conclude that diabetes mellitus and preoperative anemia are two risk factors for early and late-onset postoperative pulmonary complications, respectively. Hypoxemia and airway obstruction in Post-Anesthesia Care Unit increased four times the risk of the development of pneumonia and atelectasis at 30 postoperative days.

## Introduction

Pulmonary complications continue to be the most common adverse event after surgery^1,2^. Many surgical, anesthetic, and preoperative factors are involved in their onset; the use of neuromuscular blocking (NMB) agents and the occurrence of residual neuromuscular block (RNMB) is a proven cause in the majority of recent studies^3–5^. However, the use of pharmacological reversal of NMB, and particularly the comparison between neostigmine and sugammadex, remains a controversial factor^6–8^.

The ‘Local ASsessment of VEntilatory management during General Anesthesia for Surgery’ LAS VEGAS risk score^9^, the ‘Assess Respiratory Risk in Surgical Patients in Catalonia’ ARISCAT risk score for postoperative pulmonary complications (POPC)^10^, and the `Lung Injury Prediction Score´ LIPS model^11,12^, are three models that identify patients at risk of developing POPC.

These three scores are composed of preoperative patient characteristics and procedure-related characteristics. However, these models do not include predictive factors for the intraoperative management of NMB agents, such as neuromuscular monitoring (NMM)^13^ or pharmacological reversal with neostigmine or sugammadex.

The primary objective of this study was to design two independent predictive models. These models will assess early POPC in the post-anesthesia care unit (PACU) and late-onset POPC after 30 postoperative days. We evaluated the possible factors such as the intraoperative history and the NMB to determine which factors are important for predicting these respiratory events. On the other hand, we intend to determine whether presenting early complications in the PACU subsequently causes patients to have other POPC, such as pneumonia or atelectasis.

## Materials and methods

### Study design and setting

This is a secondary analysis of a previous observational, prospective cohort study including patients undergone general anesthesia with neuromuscular block. The primary study was conducted at Hospital Universitario Miguel Servet in Zaragoza^14^ and patient inclusion was performed by a sequential review of cases in a recruitment period from January 2016 to December 2019. As planned, the preliminary study sample size of 714 patients was used, with at least 110 patients per group, calculated with a significant level of 5% and 95% of power.

### Ethics

This study was accepted by the research ethics committee of our institution with registration code 06/2014 and reauthorized by the Regional Research Ethics Committee of Aragón with number CAB-SUG-2019-01 and EPA19/020 as requested by regional guidelines. This study was performed in line with the principles of the Declaration of Helsinki and this report follows the STROBE reporting guideline.

### Inclusion/exclusion criteria

Eligible patients must comply with the following inclusion criteria: age over 18 years, American Society of Anesthesiologists (ASA) Physical Status I–III. The exclusion criteria were as follows: ASA IV–V, known neuromuscular disease, diabetes mellitus (DM) with diagnosed neuropathy, pregnancy or lactation, known allergy to neuromuscular-blocking drugs, cardiac surgery, and scheduled admission to the ICU with mechanical ventilation. The patients were selected before the surgery and written informed consent was obtained from all subjects.

### Patient population and anesthesia

Four cohort groups were established according to the NMB agent used and its reversal: group 1 cisatracurium with no pharmacological reversal, group 2 cisatracurium plus reversal with neostigmine, group 3 rocuronium with no pharmacological reversal, and group 4 rocuronium plus reversal with sugammadex.

The patients recruited were those who were going to receive NMB agents under general anesthesia. Neuromuscular block was performed according to the standard clinical practice chosen by the anesthesiologist in charge with cisatracurium (0.1–0.2 mg/kg) or rocuronium (0.6–1.2 mg/kg). In turn, anesthetic maintenance, use of intraoperative NMM, repeat dosing, and the spontaneous or pharmacological reversal of neuromuscular block at the end of surgery depended on the same anesthesiologist who was blinded at all times to the patient’s inclusion in the study.

The pharmacological reversal was used according to the standard clinical practice and usual department protocol in the anesthetic induction. In case of having previously received cisatracurium, neostigmine (0.03–0.05 mg/kg) with atropine (0.02 mg/kg) were administered but if rocuronium was administered, participants received sugammadex (2–4 mg/kg).

### Outcomes and definitions

The primary outcome of this analysis was the POPC, as defined in other studies, such as ARISCAT, LAS VEGAS, and PERISCOPE^9,10,15^. Early POPC were considered as presenting at least one of the following respiratory events in the PACU: upper airway obstruction, desaturation below 92%, bronchoaspiration, or need for reintubation of the patient.

Moreover, late-onset POPC were defined as presenting at least one respiratory infection-like event, such as pneumonia or atelectasis, in the 30 days after surgery.

### Measurements and data handling

Patient demographic data and comorbidities recorded are detailed in Table 1. Intraoperative data included the type of surgery, elective or emergency; the type of NMB agent; the use or not of intraoperative quantitative NMM; repeated doses of NMB agent, and the use or not of pharmacological reversal of the neuromuscular block.

**Table 1:** Homogeneity and comparison of demographic data and comorbidities between groups.

Quantitative variables (n)	Cisatracurium—No Reversor Group n = 176		Cisatracurium + Neostigmine Group n = 112		Rocuronium—No Reversor Group n = 276		Rocuronium + Sugammadex Group n = 150		P-value	
	Mean	SD	Mean	SD	Mean	SD	Mean	SD	ANOVA	Kruskal–Wallis
Age; years (714)	59.81	17.37	60.81	16.79	60.78	16.32	59.4	15.54	0.81	0.60
Weight; kg (714)	70.27	11.91	72.66	12.50	72.15	13.28	75.46	16.85	0.01*	0.11
ASA score (714)	2.10	0.69	2.16	0.71	2.11	0.67	2.14	0.66	0.89	0.88

Qualitative variables (n)	Percent % (n)		Percent % (n)		Percent % (n)		Percent % (n)		CHI2	Fisher
Male (474)	23.2% (110)		16.2% (77)		40.1% (190)		20.4% (97)		0.49	0.47
Female (240)	27.5% (66)		14.5% (35)		35.8% (86)		22.1% (53)			
COPD (85)	21.1% (18)		16.4% (14)		49.4% (42)		12.9% (11)		0.09	0.09
OSAS (32)	40.6% (13)		3.1% (1)		34.3% (11)		21.8% (7)		0.07	0.06
Restrictive lung disease (8)	12.5% (1)		25% (2)		25% (2)		37.5% (3)		0.49	0.46
Asthma (14)	35.7% (5)		0% (0)		42.8% (6)		21.4% (3)		0.38	0.34
AMI (67)	22.3% (15)		23.8% (16)		35.8% (24)		17.9% (12)		0.28	0.30
Heart failure (20)	20% (4)		15% (3)		40% (8)		25% (5)		0.94	0.96
High blood pressure (329)	26.4% (87)		17.6% (58)		37.9% (125)		17.9% (59)		0.16	0.15
Anemia (62)	30.6% (19)		17.7% (11)		35.4% (22)		16.1% (10)		0.54	0.53
Chronic renal failure (40)	37.5% (15)		17.5% (7)		27.5% (11)		17.5% (7)		0.20	0.22
DM (131)	23.6% (31)		21.3% (28)		36.6% (48)		18.3% (24)		0.25	0.26
Dyslipidemia (165)	24.2% (40)		18.7% (31)		37.5% (62)		19.3% (62)		0.64	0.65
Hyperthyroidism (4)	0% (0)		25% (1)		50% (2)		25% (1)		0.71	0.76
Hypothyroidism (2)	27.2% (6)		13.6% (3)		45.4% (10)		13.6% (3)		0.80	0.86
Hepatitis (20)	35% (7)		20% (4)		20% (4)		25% (5)		0.36	0.29
Dementia (2)	0% (0)		0% (0)		0% (0)		100% (2)		0.05	0.06
Fragility (114)	20.1% (23)		17.5% (20)		43.8% (50)		18.4% (21)		0.42	0.43

Basic descriptives and tests for the demographic and comorbidities data for each group. All variables have no significant relationship with the groups. In other words, for each of these variables, there was no significant difference between the proportion of patients that were assigned to each type of neuromuscular blocking agent and pharmacological reversal. Quantitative variables: mean and standard deviation (SD) for each group, along with comparing means tests (ANOVA or Kruskal–Wallis). Qualitative variables: quantity or absolute frequency (n), proportion or relative frequency (%—percent) for each group, along with independence tests (Chi2 or Fisher). *Significance defined as p-value < 0.05. *ASA* American Society of Anesthesiologists score, *COPD* Chronic Obstructive Pulmonary Disease, *OSAS* Obstructive Sleep Apnea Syndrome, *AMI* Acute Myocardial Infarction, *DM* Diabetes Mellitus.

For the identification of early POPC, continuous clinical monitoring was performed throughout the patient's stay in the PACU by the research staff, who was blinded and was not involved in the intraoperative care of the patient. In addition, RNMB, defined as a TOF ratio < 0.9, was measured in 100% of the patients at admission to the PACU using a single TOF measurement with an intensity of 40 mA using a TOF-Watch-SX^®^ acceleromyography device [Organon, Oss, The Netherlands] calibrated in the operating room before the first dose of NMB.

In the case of late-onset POPC, the patient's electronic clinical history was consulted, noting any clinical events, laboratory tests, radiological studies, or reports of consultation to primary care or emergency care services during hospital admission or 30 days after surgery.

### Statistical analyses

To perform data analysis, a descriptive analysis was completed using the mean, standard deviation, and quartiles to summarize quantitative data. For qualitative data, frequency and percentages were used. Different inference techniques were used to detect the relationship between variables. For qualitative variables, a Chi-squared test and a Fisher’s test were used, and when proportions were compared for different groups, a difference in proportions test was used. A Kruskal–Wallis test and an ANOVA test were used to study the relationship between a qualitative variable in a quantitative variable.

To predict the occurrence of early and late-onset POPC, two independent logistic regression models were carried out using all the comorbidities (Table 1), patient history, and intraoperative variables described above. For this, the Likelihood-Ratio Test was used to select the variables of the Binomial Generalized Linear Model with logit link that were part of the final models. The modeling process was carried out in stages, eliminating in each stage the variables with a lower significance or equivalently with a higher p-value for the Likelihood-Ratio Test. All variables with a positive coefficient estimate contribute to increasing the incidence, while those with a negative coefficient decrease the incidence.

For all analyses, a statistically significant result is assumed if p < 0.05. The analysis has been developed with R version 3.4.4 (R Foundation for Statistical Computing, Vienna, Austria).

### Ethics approval and consent to participate

The study was first approved by the Ethical and Research Committee of Miguel Servet University Hospital, Zaragoza, Spain, with registration code 06/2014 (Chairperson J.M. Larrosa Poves) and subsequently it was reauthorized by the Regional Ethics Committee of Aragón (CEICA), with number CAB-SUG-2019-01 (Chairperson M. Gonzalez Hinjos) as requested by regional guidelines. Written informed consent was obtained from all subjects.

## Results

During the data collection period, 735 patients were recruited, 21 of whom were excluded for the following reasons: 18 patients for missing information and 3 patients for unexpected admission to the Surgical Intensive Care Unit. Finally, a total of 714 patients were included in the study.

Patients were divided into four cohort groups: group 1 cisatracurium with no pharmacological reversal n = 176 (24.64%), group 2 cisatracurium reverted with neostigmine n = 112 (15.68%), group 3 rocuronium with no pharmacological reversal n = 276 (38.65%), and finally, group 4 rocuronium reverted with sugammadex n = 150 (21.03%). The groups were homogeneous, and there were no differences between the groups in patient demographic characteristics or comorbidities (p > 0.05) (Table 1).

The percentage of patients who underwent NMM intraoperatively was 30.3% (n = 216), with no statistically significant differences between groups (p = 0.98). However, the incidence of total RNMB was 23.3% (n = 202), with statistically significant differences between groups p < 0.001. The observed incidence of RNMB between groups according to NMM is detailed in Table 2.

**Table 2:** Observed incidence of residual neuromuscular blockade, early and late postoperative pulmonary between groups if exists neuromuscular monitoring.

	Cisatracurium—No Reversor Group n = 176		Cisatracurium + Neostigmine Group n = 112		Rocuronium—No Reversor Group n = 276		Rocuronium + Sugammadex Group n = 150	
	No NMM	Yes NMM	No NMM	Yes NMM	No NMM	Yes NMM	No NMM	Yes NMM
BNMR	58 (44.96%)	1 (2.12%)	23 (31.94%)	11 (27.50%)	93 (42.27%)	6 (10.71%)	4 (5.19%)	4 (5.47%)
Early POPC	38 (29.45%)	9 (19.14%)	14 (19.44%)	7 (17.50%)	29 (13.18%)	5 (8.92%)	5 (6.49%)	2 (2.73%)
Late POPC	10 (7.75%)	3 (6.38%)	8 (11.11%)	2 (5%)	26 (11.81%)	1 (1.78%)	2 (2.59%)	2 (2.73%)

Qualitative variables: quantity or absolute frequency (n), proportion or relative frequency (%—percent) for each group, along with independence tests (Chi2). *Significance defined as p-value < 0.05. *RNMB* Residual neuromuscular blockade, *POPC* postoperative pulmonary complications, *NMM* neuromuscular monitoring.

### Early pulmonary complications

Concerning the first of the questions, we found that 15.27% (n = 109) of patients presented some type of early complication. In the total sample, the percentage of hypoxemia was 10.92% (n = 78), while the percentage of obstruction was 4.34% (n = 31). There were no cases of bronchoaspiration or reintubation in the PACU. The detailed observed incidence of early complications by group and according to intraoperative NMM is detailed in Table 2.

### Late-onset pulmonary complications

The incidence of late-onset POPC was 8.12% (n = 58). Among all the participants, the incidence of pneumonia was 1.68% (n = 12), while atelectasis was presented at 6.44% (n = 46). Agreeing with these outcomes, the incidence of late-onset complications observed depending on the intraoperative NMM is detailed in Table 2.

### Predictive logistic regression models for early and late-onset pulmonary complications

Of all comorbidities, only DM showed a positive coefficient estimation. Thus, this predictive model considered that DM increases the incidence of early POPC. However, the type of blocking agent, intraoperative NMM, and pharmacological reversal present a negative coefficient estimate and therefore decrease the incidence of respiratory events in the PACU (Tables 3 and 4).

**Table 3:** Variables and coefficients of the generalized linear model with likelihood-ratio test to predict early postoperative pulmonary complications (hypoxemia and airway obstruction).

	Estimate	Std. error	Z value	Pr( >|z|)
(Intercept)	− 0.98	0.17	− 5.52	< 0.001*
DM	0.72	0.24	2.90	0.003*
Rocuronium	− 1.11	0.21	− 5.04	< 0.001*
NMM	− 0.49	0.26	− 1.89	0.057
Reversal	− 0.63	0.24	− 2.59	0.009*

The table shows the model coefficients, the standard error (Std. Error), the corresponding Z value, and related P values. The most interesting thing is the sign of the coefficients of each of the variables since it indicates the direction of the influence in the response. *DM* Diabetes Mellitus, *NMM* neuromuscular monitoring; *Significance defined as Pr( >|z|) < 0.05.

**Table 4:** Probability of early postoperative pulmonary complications (hypoxemia and airway obstruction) according to diabetes mellitus, neuromuscular blockade, neuromuscular monitoring and reversal (logistic regression model).

NMB agent	Intraoperative NMM	Pharmacological reversal	DM	Probability early POPC (%)
Cisatracurium	No	No	No	27.2
Cisatracurium	No	No	Yes	43.4
Rocuronium	No	No	No	11.1
Rocuronium	No	No	Yes	20.3
Cisatracurium	Yes	No	No	18.5
Cisatracurium	Yes	No	Yes	31.9
Rocuronium	Yes	No	No	7.02
Rocuronium	Yes	No	Yes	13.4
Cisatracurium	No	Neostigmine	No	16.5
Cisatracurium	No	Neostigmine	Yes	28.9
Rocuronium	No	Sugammadex	No	6.15
Rocuronium	No	Sugammadex	Yes	11.8
Cisatracurium	Yes	Neostigmine	No	10.7
Cisatracurium	Yes	Neostigmine	Yes	19.8
Rocuronium	Yes	Sugammadex	No	3.84
Rocuronium	Yes	Sugammadex	Yes	7.59

According to the previous model, the table shows the incidences of residual neuromuscular blockade depending on the use of the neuromuscular blocking drug, the neuromuscular monitoring, and the reversal drug. *NMB* Neuromuscular blockade, *NMM* neuromuscular monitoring, *RNMB* residual neuromuscular blockade.

In the case of late-onset POPC such as pneumonia or atelectasis, of all the preoperative antecedents, only anemia, with a positive coefficient estimate, has a significant effect on our model. Likewise, intraoperative NMM, the type of blocking agent, and its reversal, with a negative coefficient estimate, decrease the incidence of late-onset POPC (Tables 5 and 6).

**Table 5:** Variables and coefficients of the generalized linear model with likelihood-ratio test to predict late postoperative pulmonary complications (pneumonia and atelectasis).

	Estimate	Std. error	z value	Pr( >|z|)
(Intercept)	− 2.25	0.25	− 8.75	< 0.001*
Rocuronium	− 0.08	0.29	− 0.30	0.75
Reversal	− 0.37	0.32	− 1.15	0.24
Anemia	0.98	0.38	2.56	0.010*
NMM	− 0.91	0.39	− 2.28	0.022*

The table shows the model coefficients, the standard error (Std. Error), the corresponding Z value, and related P values. The most interesting thing is the sign of the coefficients of each of the variables since it indicates the direction of the influence in the response. *NMM* Neuromuscular monitoring. *Significance defined as Pr( >|z|) < 0.05.

**Table 6:** Probability of late-onset postoperative pulmonary complications (pneumonia and atelectasis) according to anemia, neuromuscular blockade, neuromuscular monitoring and reversal (logistic regression model).

NMB agent	Intraoperative NMM	Pharmacological reversal	Anemia	Probability late POPC (%)
Cisatracurium	No	No	No	9.52
Rocuronium	No	No	No	8.78
Cisatracurium	No	Neostigmine	No	6.72
Rocuronium	No	Sugammadex	No	6.18
Cisatracurium	No	No	Yes	22.1
Rocuronium	No	No	Yes	20.5
Cisatracurium	No	Neostigmine	Yes	16.2
Rocuronium	No	Sugammadex	Yes	15.1
Cisatracurium	Yes	No	No	4.05
Rocuronium	Yes	No	No	3.72
Cisatracurium	Yes	Neostigmine	No	2.81
Rocuronium	Yes	Sugammadex	No	2.57
Cisatracurium	Yes	No	Yes	10.2
Rocuronium	Yes	No	Yes	9.40
Cisatracurium	Yes	Neostigmine	Yes	7.21
Rocuronium	Yes	Sugammadex	Yes	6.63

The table shows, according to the previous model, the incidence of residual neuromuscular blockade for all the possibilities of the neuromuscular blocking drug, neuromuscular monitoring, and reversal drug. *NMB* Neuromuscular blockade drug, *NMM* neuromuscular monitoring, *RNMB* residual neuromuscular blockade.

### Reversal with neostigmine or sugammadex and pulmonary complications

Regarding the neuromuscular reversal and POPC in the PACU, we saw that the incidence of these complications when cisatracurium was used without pharmacological reversal was 26.70% (n = 47), while if it was reversed with neostigmine it was 18.75% (n = 21), with no statistically significant differences between groups (p = 0.12). If rocuronium was not reversed, the incidence of early complications was 12.32% (n = 34), while if we revert with sugammadex it was 4.67% (n = 7), with statistically significant differences between them (p = 0.01).

**Figure 1:**
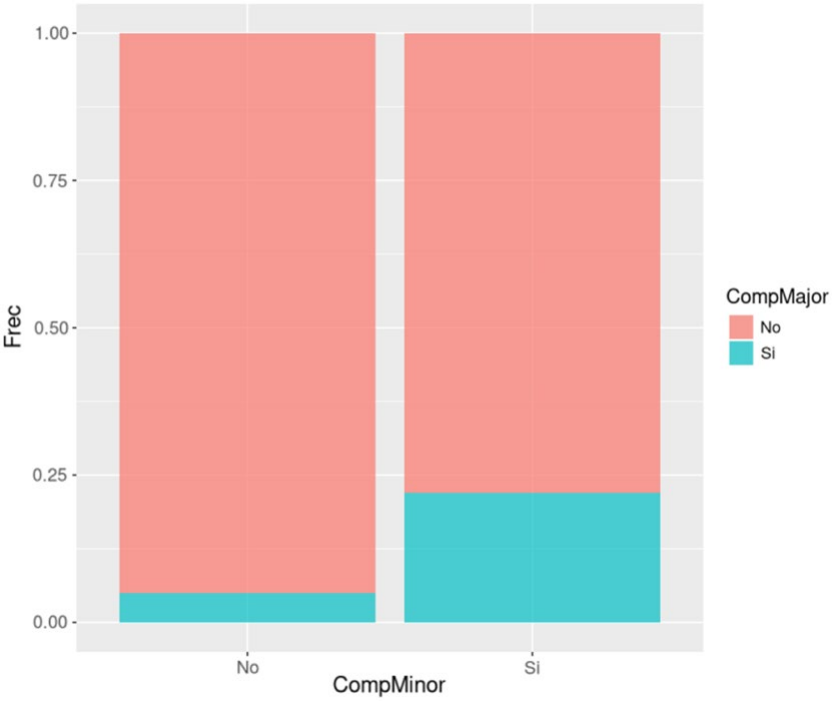
Comparison of early and late-onset postoperative pulmonary complications by groups. *CompMinor* early postoperative pulmonary complications, *CompMajor* late-onset postoperative pulmonary complications.

The incidence of late-onset complications such as pneumonia or atelectasis observed in the cisatracurium group without reversal was 7.39% (n = 13). Regarding the group using neostigmine, this incidence was 8.93% (n = 10). However, we did not observe any statistically significant differences between both groups (p = 0.637). On studying rocuronium with no pharmacological antagonism, the incidence of late-onset complications was 9.78% (n = 27), and if we reverse with sugammadex, the incidence decreases to 2.67% (n = 4), statistically significant with p = 0.006 (Fig. 1).

In this particular case, the reversal of rocuronium with sugammadex decreases the incidence of hypoxemia (p = 0.012) and atelectasis (p = 0.028), but not that of obstruction (p = 0.45) or pneumonia (p = 0.24).

### Early pulmonary complications and their relationship to late-onset complications

**Figure 2:**
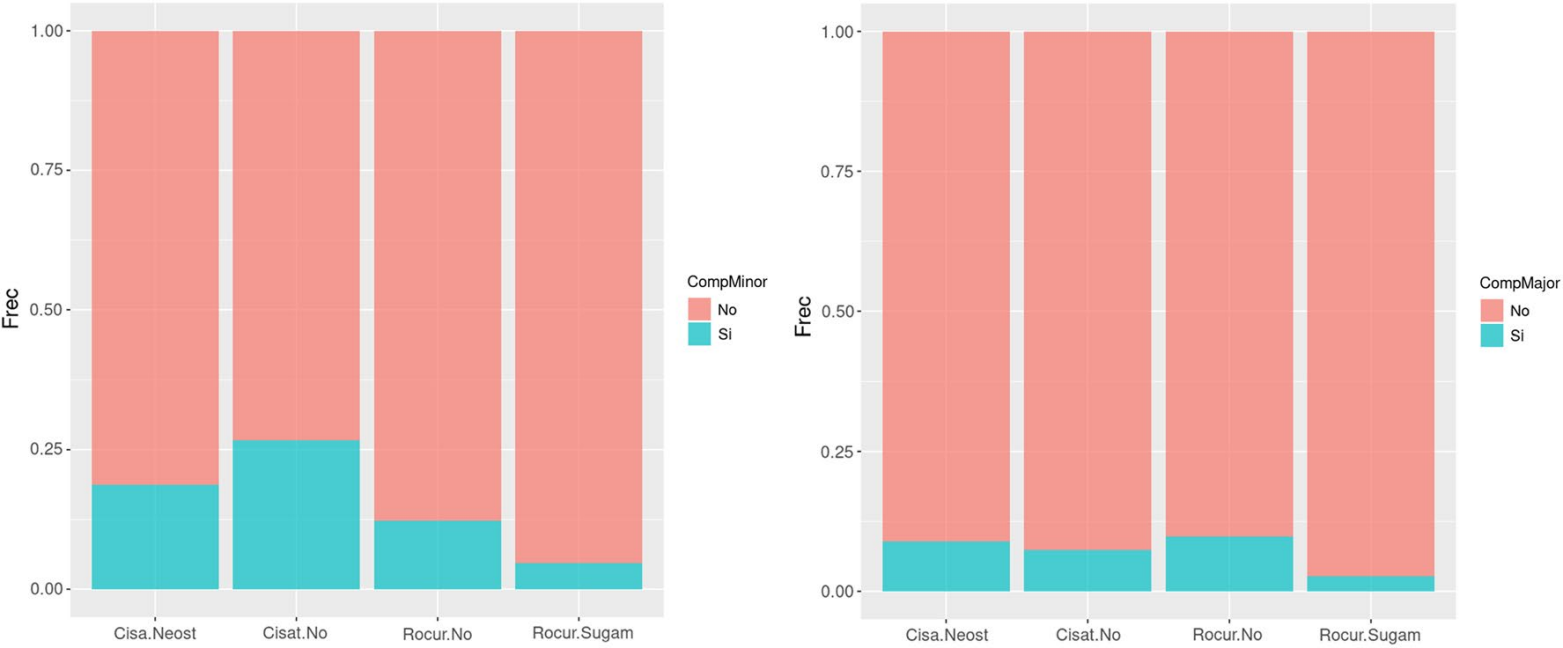
Comparison of late-onset postoperative pulmonary complications (pneumonia and atelectasis) if exits early postoperative pulmonary complications (hypoxemia and airway obstruction). *CompMinor* early postoperative pulmonary complications, *CompMajor* late-onset postoperative pulmonary complications.

In our patient sample, the incidence of a late-onset POPC if we have not previously had any early complication was 4.96% (n = 30). However, if the patient has previously had any type of early complication the incidence of a late-onset POPC was 22.02% (n = 24), and therefore the risk of suffering other late-onset complications was four times higher (Fig. 2). However, these statistical differences were only for atelectasis (p < 0.001) but not for pneumonia (p = 0.079).

If airway obstruction occurs, the risk of atelectasis increases from 6.88 to 22.58% (p = 0.002), and if hypoxemia occurs, the incidence increases from 5.82 to 21.79% (p < 0.001).

## Discussion

This secondary analysis of a prospective, observational study provides two independent predictive models. These models have determined that DM and anemia are the only preoperative factors capable of predicting the risk of developing POPC, and that pharmacological reversal with sugammadex, but not with neostigmine, is significant to decrease the incidences of hypoxemia and atelectasis in the immediate and late postoperative period.

**Figure 3:**
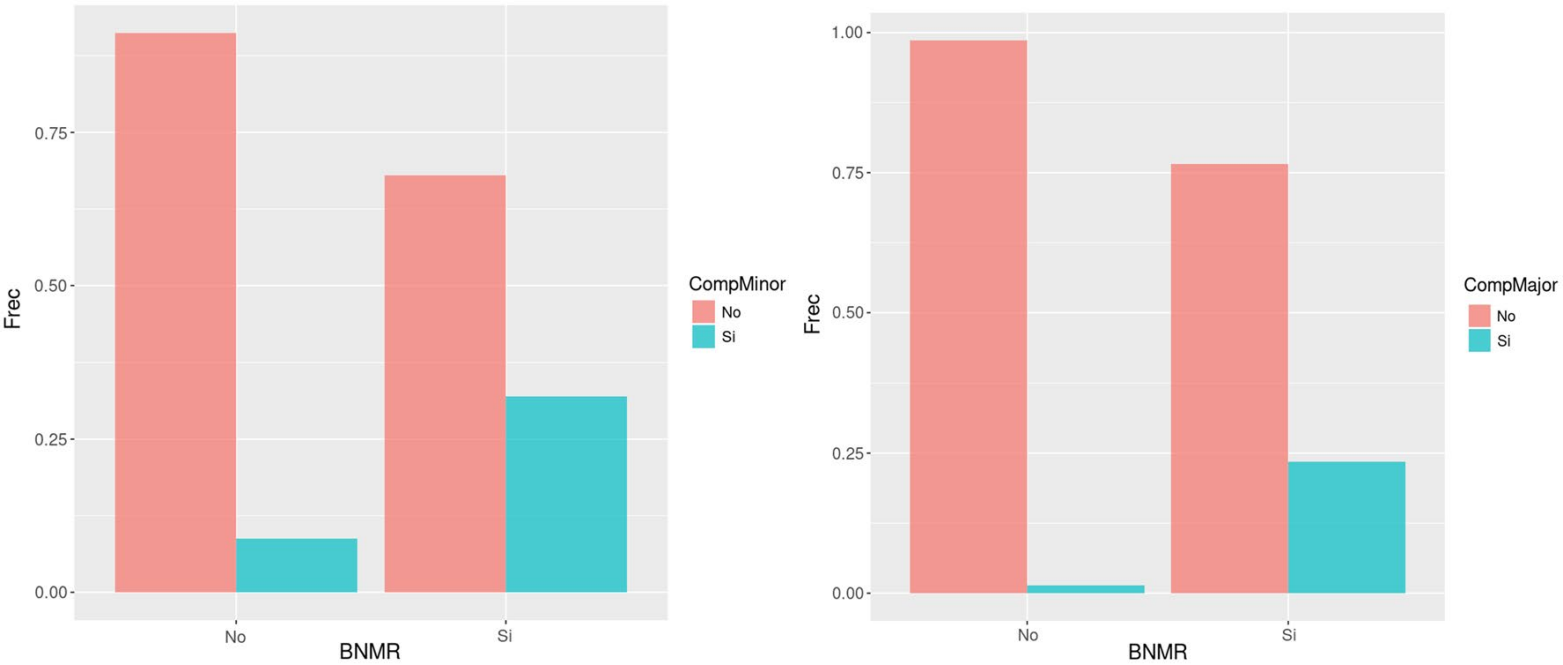
Comparison of early and late-onset postoperative pulmonary by the residual neuromuscular blockade. The figure illustrates the incidence of early and late-onset complications in our sample of patients, classified by the presence or absence of RNMB. Patients with RNMB had an incidence of early POPC of 32% and an incidence of late-onset complications of 23.50%. Patients without RNMB had an incidence of early complications of 8.75% and an incidence of late-onset complications of 1.36%. *BNMR* residual neuromuscular blockade, *CompMinor* early postoperative pulmonary complications, *CompMajor* late-onset postoperative pulmonary complications.

In the first study^14^, we calculated a predictive model for RNMB and demonstrated its relationship with postoperative pulmonary complications (Fig. 3). In contrast, this second analysis focuses on the predictive model for postoperative pulmonary complications and shows how early pulmonary complications can lead to late-onset complications.

In the case of early complications in the PACU, as can be seen in the results, the use of rocuronium and its reversal with sugammadex, as well as intraoperative monitoring, significantly decreases the risk of developing early complications. On the other hand, DM was the only comorbidity that appears to increase the risk in our patient sample.

In this regard, we can see that if a patient presents DM and we also use cisatracurium with no intraoperative NMM or pharmacological reversal, the probability of having some type of early complication in the PACU is 43.4%. However, if we do perform intraoperative NMM in the same patient and use rocuronium-sugammadex, the incidence of respiratory events in the PACU is 7.5%; that is, it decreases by 35.9%.

On the other hand, the important factors in the occurrence of late-onset complications, such as pneumonia and atelectasis, were anemia, intraoperative NMM, and the type of blocking agent-reverting agent. Therefore, if a patient is anemic, with no intraoperative NMM and no pharmacological reversal, the probability of suffering POPC in the 30 days after surgery is 22.6%. However, this incidence decreases to 3.8%, in the case of monitoring and pharmacological reversal with sugammadex.

Unlike that shown in other studies^10,16,17^, in our models, the ASA index^18^ and other comorbidities, such as obstructive sleep apnea^19^, were not capable of predicting an increased risk of this type of respiratory event.

DM and metabolic syndrome have been widely reported as independent risk factors for an increased risk of POPC in the postoperative stage in previous studies^20^ and meta-analyses^21,22^.

It should be noted that many patients with DM have specific neurological and neuromuscular dysfunctions^20–22^, though diabetic patients with diagnosed neuropathy were excluded from our study from the start. It has also been shown that subjects with DM and even with prediabetes have reduced respiratory drive under hypoxic conditions, lower forced vital capacity (FVC), lower forced expiratory volume in one second (FEV1), as well as a greater percentage of restrictive spirometric patterns as compared to non-diabetic patients^23–25^.

Moreover, preoperative anemia has already been described in other predictive models such as the ARISCAT score^9^, LAS VEGAS score^10^, and in several studies^3,26^ as an independent risk factor for the development of POPC.

### Late-onset complications dependent on early complications

Nevertheless, we also took a special interest in the association between early complications and the development of late-onset complications. As seen in the results, the risk of suffering atelectasis in the late postoperative period was four times higher in the event of an episode of obstruction or hypoxemia in the immediate postoperative period in the PACU.

The association between postoperative desaturation and the occurrence of atelectasis is due to airway closure and the subsequent decrease in functional residual capacity^27,28^. In fact, according to Alday et al. and Duggan et al., atelectasis may occur in up to 90% of anesthetized patients and should be suspected when oxygenation is affected after surgery^29,30^.

We have already seen that RNMB was related to all POPC, as can be seen in Table 2, in over 90% of the cases in which there was no RNMB, there were no complications of any kind.

### Pharmacological reversal and postoperative pulmonary complications

Concerning pharmacological reversal and the onset of POPC, we see that reversal with neostigmine does not only not significantly decrease their incidence, but also that in the case of late-onset complications, like pneumonia and atelectasis, it appears to increase it. In contrast, pharmacological reversal with sugammadex decreases the risk of developing hypoxemia in the PACU by three times and the risk of developing atelectasis over the 30 days after surgery by four times.

These results are consistent with data from the study by Krausse et al.^31^ or the STRONGER study by Keterpal et al.^32^, a multicenter study with 30026 patients that showed that the administration of sugammadex was associated with a 30% reduction in the risk of POPC as compared to neostigmine.

In contrast, Togioka et al.^16^ have shown that although reversal with sugammadex does reduce RNMB by 40%, they found no differences in the development of POPC when compared to neostigmine. Nevertheless, it should be taken into account that, in that study, the definition of hypoxemia was stricter, since it was defined as patients with oxygen saturation below 90%, unlike the criteria of our study or ARISCAT and LAS VEGAS where it was defined as being below 92%^4,9,10^.

However, while some studies, such as Togioka et al.^16^, Abola et al.^28^ and Alday et al.^29^, have raised controversy, along with other research such as POPULAR study by Kirmeier et al.^33^, the majority of recent trials have concluded that sugammadex reduces the incidence of POPC^34–37^.

Oh et al.^38^ also concluded that the incidence of unplanned readmission at 30 days post-op was 34% lower in patients receiving sugammadex as compared to those who received neostigmine, with the potential economic benefit involved.

Furthermore, in line with our recommendations, the recently published international guidelines, such as those from the European Society of Anaesthesiology and Intensive Care (ESAIC)^39^ and American Society of Anesthesiologists (ASA)^40^ task forces, emphasize the importance of using both neuromuscular monitoring and pharmacological reversal to minimize RNMB and other complications associated with muscle relaxants during the postoperative period.

### Limitations

This study has certain limitations. First, this is a single-center observational study^14^ that may have certain statistical power limitations for the detection of potential differences compared to the capacity of other multicenter randomized clinical trials. Moreover, there is no consistency in the literature to define a postoperative pulmonary complication. Therefore, the comparison of the outcomes reported is controversial. In addition, we have not analyzed factors such as duration of surgery, location of the surgical incision, ventilation parameters, recruitment maneuvers, and possible analgesic techniques that may contribute to an increase in these complications. In future studies, we will collect data providing information on hospital stay, the potential economic effect, and the reduction of healthcare costs that these preventive measures may entail^41,42^.

## Conclusion

In conclusion, based on the results of our predictive models, it may be concluded that DM and preoperative anemia are two important risk factors for the development of early and late-onset POPC, respectively.

Moreover, hypoxemia and airway obstruction in the PACU affects the subsequent development of pneumonia and atelectasis at 30 postoperative days, so we highly suggest that the best prevention is to minimize the RNMB through intraoperative NMM and the use of rocuronium with pharmacological reversal with sugammadex.

## Data Availability

The datasets used and analyzed during the current study are available from the corresponding author upon reasonable request.

## Abbreviations

POPC: Postoperative pulmonary complications
NMM: Neuromuscular monitoring
NMB: Neuromuscular blockade
RNMB: Residual neuromuscular blockade
PACU: Post-anaesthesia care unit
TOF: Train of four
